# Every School Healthy: An Urban School Case Study

**DOI:** 10.1111/josh.12965

**Published:** 2020-11-12

**Authors:** Sue Baldwin, Assunta R. C. Ventresca

**Affiliations:** ^1^ Buffalo Public Schools Office of Whole Child Initiatives 58 Lantern Ln. Amherst NY 14211 USA; ^2^ Buffalo Public Schools 84 Lorraine Avenue Buffalo NY 14220 USA

**Keywords:** whole school, whole community, whole child (WSCC) model, whole child, sexual health education, special needs students, English as a new language (ENL) students, community health workers

## Abstract

**BACKGROUND:**

In this case study, multiple participants in a large urban school district used the Whole School, Whole Community, Whole Child (WSCC) model to guide development of a district wellness policy. The model's health education component is highlighted, focusing on concerns for special needs students and ones speaking English as a New Language (ENL).

**METHODS:**

Organizational structure was developed around the WSCC model and district wellness policy implementation through coordination, collaboration, and communication (3Cs) of programs, policies, and processes/practices (3Ps).

**RESULTS:**

The WSCC approach guided the creation of a district wellness policy that influenced programming for students with special needs and required Youth Risk Behavior Survey (YRBS) data collection. Using YRBS and School Health Index (SHI) data in planning sexual health education illustrated improvements over time. Formation of the *School Health and Wellness Collaborative* under WSCC improved family engagement in sexual health education programs and practices. Relationships were established with community partners to impact student's sexual risk behaviors. Finally, the district co‐created and implemented an evidenced‐based sexual health curriculum, modifying it for special education and ENL students.

**CONCLUSIONS:**

The WSCC approach is system changing. It takes time to develop the relationships vital to improve the 3Cs and 3Ps. Success is enhanced with a district wellness coordinator, the right people at the table, valid health data, and administrative and board support.

This urban school district case study demonstrated the power of using the Whole School, Whole Community, Whole Child (WSCC) model, a strong equitable wellness policy, and Youth Risk Behavior Survey (YRBS) and School Health Index (SHI) data to support the improvement of health and education outcomes for students. This paper examines comprehensive health education, specifically comprehensive sexual health education focusing on the district's concern for students with special needs and students who speak English as a New Language (ENL).

We examined Buffalo Public Schools, the second largest school district in New York State. Demographics outlined from the *US Census Bureau* ^1^ showed that almost half of the district's children lived in poverty which ranks it the fourth worst among all the nation's major cities. As of 2018, the district supports 33,415 students, of which 35% are ENL and 21% are students with disabilities. Approximately 77% are considered to be economically disadvantaged and 96% of the district population is classified as Title I. The racial distribution of the population is 45% black, 21% Hispanic, 20% white, 10% Asian, and 4% “other.” Over 82 different languages are spoken by students in this district. For the 2017‐2018 school year, the district's 4‐year graduation rate was 64%.^2^ The demographics led the *Whole Child Advisory Board* into discussion and concerted efforts to ensure equity in wellness policy development along with adaptation of the comprehensive sexual health education curriculum.

In this district, the *Whole Child Advisory Board, Whole Child District Health Committees*, and school‐level *Whole Child Well‐Being Teams* are managed by the *Office of Whole Child Initiatives* led by a seasoned District Wellness Coordinator as illustrated by the *WSCC Organizational Chart* (Figure 1). This District Wellness Coordinator assessed, planned, and evaluated the implementation of and reported progress on the wellness policy titled *Making Health Academic*. The District Wellness Coordinator directed the administration, analysis and reporting of YRBS and SHI (assesses all WSCC domains) data collection across the district. The Coordinator annually led all 60 schools' *Whole Child Well‐Being Teams*, 13 district *Whole Child Health Committees*, and the *Whole Child Advisory Board*. All teams and committees use YRBS and SHI data to create specific, measurable, attainable, realistic, and timely (SMART) committee goals to decrease student health risk behaviors. The WSCC vision, mission, roles, responsibilities, organizational chart (Figure 1), and the wellness policy built the WSCC model infrastructure and fostered its sustainability.^3^ The coordinator ensured fidelity of implementation of the WSCC model across the district and all of its schools.

**Figure 1:**
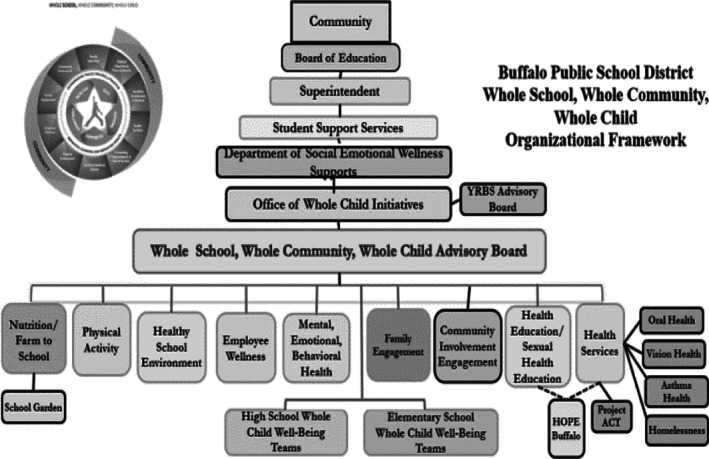
Office of Whole Child Initiatives: WSCC Organizational Chart

There are 60 schools: elementary schools supporting students in grades PK‐8, a few schools with students in grades 5‐12, and 19 high schools that accommodate students in grades 9‐12. The district is fortunate, unlike many school districts, to have a full‐time nurse at each building. Nearly all schools have a full‐time school psychologist, counselor, and social worker based on school enrollment and need. All district and school Whole Child Well‐Being Committees and Teams begin their name with “whole child.” For reader ease and readability, we have omitted the “Whole Child” wording from their titles for the remainder of this paper.

## Needs Assessment and District Response

This section focuses on comprehensive sexual health education for all students with consideration for students with special needs and ENL students. We will outline how the district assessed the need for sexual health education and how the district addressed those needs through the WSCC model. Students, parents, community partners, and district personnel co‐created and supported comprehensive sexual health education. We also will review the successes of this district's wellness policy, the data that drove the changes and continual professional development across WSCC domains that supported sexual health education.

## Wellness Policy Success

**Figure 2:**
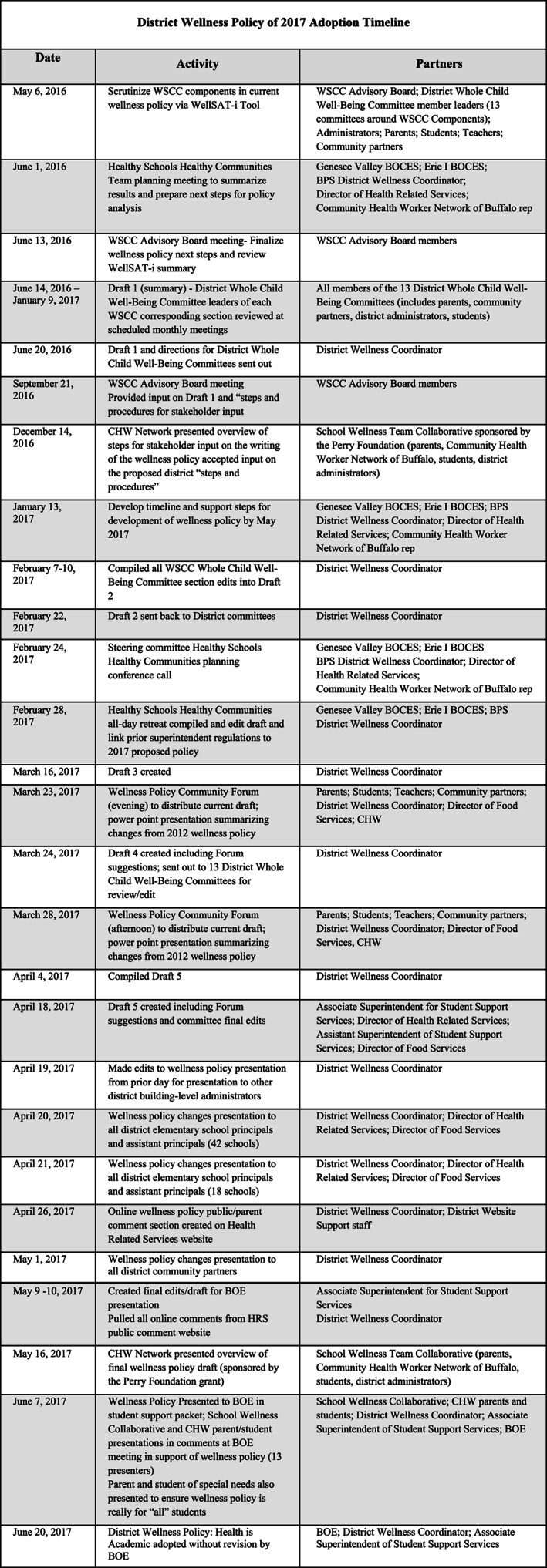
2016–2017 District Wellness: Health is Academic Policy Adoption Timeline

The strength of the wellness policy is driven by the leadership of the District Wellness Coordinator and implementation across all of the WSCC model domains (ie, parent and community engagement, school guidance, school psychologist and social workers, health‐related services, health education, and physical education). The wellness policy not only stressed coordination, collaboration, and communication (3Cs) of all district policies, programs, and practices (3Ps), but also was created through the continual use of 3Cs and 3Ps; this has become our standard of practice. Figure 2 showcases all the steps taken to accomplish wellness policy updates via the 3Cs with parents, students, community partners, and school personnel. We recognize that school districts can be successful in creating, adopting, and implementing a WSCC approach‐based wellness policy with these essential elements: administrative support, a wellness champion, and engaged parent and community partners in which health promotion “fits.”^4^ A district wellness champion^4^ could be a physical educator, health teacher, nurse, school administrator, parent, or anyone who has a passion to create a healthier school environment. Data is an important component and it can drive action. This case study occurred over a period of 7 years and the work continues to become institutionalized across the district and city. Research impresses that schools in districts with strong policies are more likely to implement the policy and associated practices.^5^, ^6^ The strength of the policy lies in strong language to indicate that action is required and includes words such as shall, will, must, require, all, comply, and enforce.^6^ The policy has clearly stated goals that are able to be enforced via the District Wellness Coordinator, district whole child health committees and all 60 *School Well‐Being Teams*. The district wellness policy stressed the importance of district and school responses to sexual health education through the WSCC model. Numerous district and community partner actions resulted in positive student sexual health behavior change. The district activities include adopting a sexual health curriculum, curriculum adaptations for students with special needs and ENL students, condom availability program policy adoption, professional development, parent engagement and education opportunities, and the formation of the *Student Engagement Committee*. The Erie County Department of Health (DOH), community partners, and student groups worked collaboratively to support all students. The wellness policy stated that sexual health education be taught in a planned, sequential, and age‐appropriate manner. The policy also impressed that this education be medically accurate and culturally and linguistically responsive to student learning styles. Finally, sexual health education should be evidence‐based or grounded in research, aligned with state and/or national standards, and include the condom availability program. The condom availability program student education began in the grade 7 health courses and upon completion the students become eligible for accessing available no cost condoms in grade 9.

The District Wellness Coordinator fostered trusting relationships with parent leaders, including ENL and parents of students with special needs, who have been a driving force for strengthening wellness policy development and implementation. The wellness policy language addressed students with disabilities and those with language barriers. The continuous use of the 3Cs, with over 275 community partners, led to data driven wellness policy development coordinated with a variety of partners (Figure 2). It took a year to redesign the policy around the WSCC model using the *WellSAT 3.0*.^7^ District *Whole Child Health Committees* formed health needs arose and initially reflected the 10 components of the WSCC model. Creating a wellness policy using the WSCC model formalized the development of specific committees. School district departments took leadership roles on these committees. For example, the Department of Food Service became highly engaged to address the obesity issue in our district, the Parent Engagement Office integrated WSCC language into their department policies, and the Department of Health and Physical Education formed the *Comprehensive Health Education Committee* to implement a stronger health curriculum and address risky sexual health behaviors. The *Whole Child Advisory Board* is composed of district and community partner co‐leaders from all 16 *District Health Committees*. The *Comprehensive Health Education Committee* was formed to assess health education needs and to identify solutions. This committee reviewed data and recognized that comprehensive health education, including age‐appropriate sexual health education enables students to master the essential knowledge, attitudes, and skills to adopt and maintain life‐long healthy sexual behaviors. The *Advisory Board* assessed the policy every 3 years using the most current *WellSAT* tool. Once all WSCC domains have been assessed for clarity, language, and comprehensiveness, *District Health Committee* leaders then used the assessment results with their committee to revise their designated WSCC domain of the policy. We hosted many parent, student, and large community partner meetings for these stakeholders to provide feedback on each WSCC domain of the policy. All *School Well‐Being Teams* completed a wellness policy feedback activity in their school at a faculty meeting to seek feedback from all school‐level staff. The *Advisory Board* reconvened to review and edit the feedback from all stakeholders on all domains to finalize the policy. Feedback, through collaboration and communication with the *Special Education Parent Advisory Committee* (SEPAC), impacted wellness policy language. The SEPAC chairperson was invited to sit on the *Advisory Board* as the policy was being evaluated and updated. The SEPAC chairperson advocated for wellness policy adoption at a school board meeting and now the policy addresses students with special education needs.

The policy was unanimously adopted by the board of education due to extensive use of the 3Cs (Figure 2) and its culturally and linguistically appropriate language that addressed equity issues across all policy areas, including special needs and ENL students. To be equitable every school had a *Well‐Being Team*, received their own YRBS school report, and participated in and obtained their SHI needs assessment. The district *Advisory Board* and *District Health Committees* (each representing the components of the WSCC model; Figure 1) consistently strived to include students, parents, teachers, school administrators, higher education/research partners, board of education members, health professionals, appropriate community partners, and the district medical director in their monthly meetings. All of the committees and teams that exist under the *WSCC Organizational Chart* (Figure 1) utilized data from the YRBS, the SHI, school climate surveys, county and state health reports, and any other pertinent health or educational data (ie, attendance rates, suspension rates, bullying rates) to create district and school action plans. These action plans were incorporated into the state mandated school improvement plan as part of each school's work to improve student academic and health outcomes.^9^ The wellness policy mandated development of a district‐wide plan, progress monitoring and reporting of WSCC model implementation of the policy to the board of education and community.

In 2019, the district responded to the United States Department of Agriculture^9^ requirement to update the wellness policy. A more robust policy continued to evolve utilizing the *Rudd Institute's WellSAT WSCC* ^8^ assessment. The *WellSAT WSCC* tool examined the strength of wording and comprehensiveness of policy around all domains of the WSCC model.

## Assessing Need for Comprehensive Sexual Health Education: YRBS Administration

Parents rallied and attended a Board of Education meeting to advocate for the administration of YRBS to assess student health risk behaviors and to evaluate district health programs. Strong parent leadership led to the district administering the US Centers for Disease Control and Prevention's (CDC) YRBS to all students in grades 6‐12, starting in 2011. Students complete the YRBS online in a census data collection format. The YRBS results guided the district as well as its WSCC community partners toward addressing identified student sexual health risk behaviors and toward fostering a culture of health and well‐being across the district and its schools. The wellness policy mandates the district to collect YRBS every odd numbered year and is led by the *YRBS Advisory Board* composed of higher education partners and other key research leaders in public health, social work, exercise science, nutrition, and dental medicine. The *YRBS Advisory Board* meets to evaluate, plan, lead, implement, and guide YRBS data collection.

The WSCC model and its emphasis on coordination, collaboration, and communication around a variety of programs, policies, and practices has reduced the fragmentation related to health and education in this district. As a result, all district high schools and middle schools have shown improvement of risky sexual health behaviors (Tables 1 and 2). Comprehensive sexual health education became the district's priority due to alarming data. According to the district 2011, YRBS middle school data, almost 16% of students reported “ever having sexual intercourse.” The data has trended down with only 8% of students reporting “having sexual intercourse” in 2017; a 49.4% decrease. Of students reporting that they “had sexual intercourse before age 11 years,” results reflect a 43.2% decrease. Students who had sexual intercourse with 3 or more partners' indicated a 57.6% change since 2011. Condom utilization has not reflected positive behavior change at this time (Table 1).

**Table 1:** Middle School: Grades 6‐8 Sexual Health YRBS Survey Results Over Time

Middle School Grades 6–8*					
	2011	2013	2015	2017	
N = Valid Survey Sample Size	N = 5408	N = 5854	N = 6023	N = 5868	2011–2017% Change
Ever had sex in their lifetime	15.8%	13.3%	8.4%	8.0%	−49.4%
Had sex before age 11	4.4%	4.0%	2.3%	2.5%	−43.2%
Had 3 or more sexual partners	6.6%	5.6%	2.7%	2.8%	−57.6%
Did not use condoms	21.6%	28.1%	40.9%	36.5%	69.0%

* 2011: YRBS Response rate: 76%, rates varied from school to school 94%‐21%. 2013: YRBS Response rate: 76%, rates varied school to school 94%‐25%. 2015: YRBS Response rate: 85%, rates varied from school to school 93%‐47% (only 2 schools of 55 below 60%). 2017: YRBS Response rate: 86%, rates varied from school to school 99%‐1% (5 middle schools of 55 below 60%).

**Table 2:** High School: Grades 9‐12 Sexual Health YRBS Survey Results Over Time

High School Grades 9‐12					
	2011	2013	2015	2017	
N = Valid Survey Sample Size	N = 5345	N = 5462	N = 5934	N = 6253	2011‐2017% Change
Ever had sex in their lifetime	51.1%	44.6%	38.3%	31.1%	−39.1%
Had sex before age 13	11.8%	10.6%	7.5%	5.5%	−53.4%
Had 4 or more sexual partners	20.3%	16.5%	12.3%	8.4%	−58.6%
Did not use condoms	30.8%	35.5%	39.5%	40%	29.9%
Recently sexually active	37.9%	33.3%	27.8%	21.6%	−43.0%
Used alcohol or drugs before last sexual activity	23.4%	24.5%	19.2%	21.1%	−9.8%
Did not use any method to prevent pregnancy	n/a	14.7%	14.7%	15.7%	6.8%
Did not use birth control pills to prevent pregnancy	89.3%	87.1%	87.1%	87.0%	−2.6%

2011: Response rate: 66%—valid surveys n/a—rates varied from school to school 90%‐25%. 2013: Response rate: 61%—valid surveys 91%—rates varied from school to school 86%‐24%. 2015: Response rate: 67%—valid surveys 97%—rates varied from school to school 84%‐17%. 2017: Response rate: 69%—rates varied from school to school 88%‐32%.

The 2011 high school YRBS data report showed that half of the students who completed the survey affirmatively responded to the question that they “ever had sexual intercourse in their lifetime.” Sexual activity for high school students has consistently declined since 2011, including over a 40% drop in the percent of district high schoolers who “have ever had sex” and the percent who “had sex recently” (within 30 days of taking the survey). In prior YRBS administrations, students, in this district, were far more likely to engage in sexual activity than New York State students, but they are now about the same likelihood. In 2017, “early sexual initiation” and students “having 4 or more partners” have both dropped by more than 50% since 2011. However, district students still engage in these risk behaviors more than New York State students. Many students are not using condoms when having sex. This was the first year since 2011 that district students are no more likely to “have recently had sex” or “had a high number of sexual partners” than NYS students, and only slightly more likely to “ever had sex.” Whereas sexual activity has declined steadily since 2011, the percent of sexually active students in 2017 who “did not use a condom the last time they had sex” has risen each year, increasing by 30% since 2011 (Table 2). On the 2017 survey, about 16% of sexually active district high schoolers did not use any method to prevent pregnancy the last time they had sex. Ongoing evaluation of YRBS and SHI data and the management of fidelity of sexual health instruction in 60 schools are essential to continued, long‐lasting changes in student sexual health knowledge, attitudes, skills, and behaviors for a lifetime.

Annually, approximately 200 students in the district are identified as pregnant or parenting teens. Many of these students are students with special needs. County data showed teen pregnancy rates in Buffalo were double (33.2 teen pregnancies per 1000 female population aged 15‐19 New York State versus 67.54 in this city) the state rates and were even higher in 9 identified zip codes in the city (73.32 teen pregnancies per 1000 female population aged 15‐19).^10^ Also alarming to district decision‐makers was that nationally, only about 50% of teen mothers receive a high school diploma by 22 years of age, whereas approximately 90% of women who do not give birth during adolescence graduate from high school.^11^ Additionally, the children of teenage mothers are more likely to have lower school achievement and to drop out of high school, have more health problems, be incarcerated at some time during adolescence, give birth as a teenager, and face unemployment as a young adult.^12^ These effects continue for the teen mother and her child even after adjusting for those factors that increased the teenager's risk for pregnancy, such as growing up in poverty, having parents with low levels of education, growing up in a single‐parent family, and having poor performance in school.^10^

The district used data to determine that there was a need to address specialized sexual health education for several groups of students. School‐level YRBS data demonstrated that special education students attending an ungraded school designed for high school students with special needs up to age 21 and students in schools with high populations of ENL learners were determined to be at greater risk for unhealthy and unsafe relationships, dating and sexual violence, and to experience sexual coercion as outlined by their school‐level YRBS data. Low condom utilization as per YRBS results and high rates of teen pregnancy led the district to believe students were unaware of community health services and that students were unfamiliar with their sexual health rights and available resources to keep them safe and healthy.

## Addressing Comprehensive Sexual Health Education Needs and Adaptations

**Figure 3:**
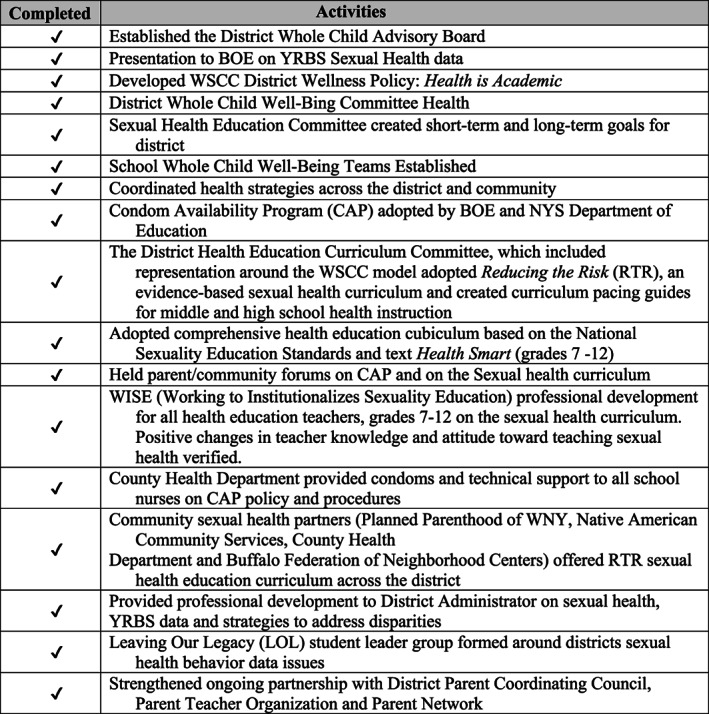
Using the WSCC Model: Steps Taken to Improve Student Sexual Health

The wellness policy addressed each domain of the WSCC model including health education. Through coordination, collaboration, and communication (3C's) with all district‐recognized parent groups, 2 student peer educator groups and all sexual health community partners, the Condom Availability Policy was adopted by the Board of Education (Figure 3). Recommended by the *Comprehensive Health Education Committee*, the adoption of a middle and high school health education curriculum, *HealthSmart*, included the condom availability policy, and an evidence‐based high school sexual health curriculum called *Reducing the Risk*. One of the partners worked with the district upon request of a building administrator at the school for students with disabilities up to age 21, to develop a sexual health education curriculum targeting students with special needs. Special education adaptations were made in collaboration with school special education staff, instructional coaches, parents, occupational therapy, and physical therapy staff by adapting the seventh grade *HealthSmart* curriculum and several high school *Reducing the Risk* lessons. Adaptations included ongoing sexual health education through the creation of interactive lessons while offering additional classes. After its implementation for 3 years, this curriculum is being examined for expansion to all schools housing students with special needs including in classrooms that typically have 6 or 8 students per teacher. Sexual health education will benefit disabled students and give them the knowledge and skills to seek out resources for healthy sexuality across their lifetime.^13^

Translation was determined to be an essential adaptation for ENL students learning sexual health education. Translators were secured for some of the high ENL population schools where community partners could not assist with translation. In addition, peer‐to‐peer translation was used to assist with sexual health education in several classrooms.

## Key Stakeholder Engagement

The *School Health and Wellness Collaborative* or family engagement domain of the WSCC model was formed to collaborate with the district to promote communication, collaboration, information sharing, shared decision‐making, and continued research on best practices for family engagement in school sexual health education. The *School Health and Wellness Collaborative* included community health worker trained parents and students, including parents of students with special needs and ENL students. These families attended and advocated for a comprehensive sexual health education curriculum and the adoption of the condom availability policy during numerous school district organized community forums. These community health workers attended, participated, and/or led their school‐based *Well‐Being Teams* and participate on the district *Comprehensive Health Education Committee*. Community health workers also engaged in wellness policy writing and advocacy efforts to ensure comprehensive sexual health education. Parents and students are highly involved in decision‐making and are empowered to participate in meaningful, open dialog. The district also recognized that parents and students have direct knowledge about the diverse family and community‐level assets and challenges that impact them around sexual health. Research has shown that parents involved in school health activities will not only impact their children's health behaviors, but also their academic achievement.^14^ The *School Health and Wellness Collaborative* recognized that engaging with families can inform, complement, reinforce, and accelerate educators' efforts to educate the whole child.

## Professional Development

Gaps found in the SHI, completed in all schools revealed that students were not receiving appropriate comprehensive sexual health education and teachers had not received professional development to teach sexual health education. Community partners, who were members of the *Comprehensive Health Education Committee*, helped address this gap, engaged in advocacy, and contributed to the success of sexual health outcomes. The Genesee Valley Educational Partnership provided professional development for teachers on the adopted sexual health curriculum, including adaptations for students with special needs and ENL students. Teachers received training on the *CDC's Characteristics of Effective Health Education*,^15^ and health instruction that incorporated the use of *HealthSmart*, *Reducing the Risk* and the condom availability program. Professional development also provided skill development on how to utilize school YRBS data to prioritize district health education pacing guides for their buildings.

Sexual health community partners from Genesee Valley Education Partnership, Planned Parenthood of Western New York, Native American Community Services, Erie County Department of Health, and the Federation of Neighborhood Centers collaborated to supplement sexual health education instruction in seventh and ninth grade health courses. These partners assisted the committee in the creation and implementation of the condom availability policy and provided professional development to all district health teachers on how to incorporate the condom availability policy into the sexual health curriculum.

Erie County Department of Health provided professional training for all district nurses on the condom availability program as it is related to the *Reducing the Risk* sexual health curriculum. Another example of the use of the 3Cs with Erie County Department of Health, Cicatelli Associates, and the district resulted in federal funding of the Teen Pregnancy and Prevention grant. This $10 million grant fostered the establishment of *H.O.P.E. Buffalo*. *H.O.P.E. Buffalo* is the community‐wide pledge for teen health—a youth and community‐led collaborative of diverse stakeholders, teens, and adults, working together to promote equitable access to high‐quality and comprehensive sexuality education and reproductive health services. *H.O.P.E. Buffalo* values *H*ealth, *O*pportunity, *P*revention and *E*ducation and believes that teens have the right to grow into the adults they want to be—whatever that means for them. *H.O.P.E. Buffalo* supported student visits to community sexual health agencies and then created the *H.O.P.E. Buffalo Pocket Guide*, a student‐friendly resource for sexual health services. *H.O.P.E. Buffalo* funds purchased *Be Proud Be Responsible* the district's grade 7 evidence‐based sexual health curriculum. The grant also funded *Raising Healthy Children*, an evidence‐based youth development curriculum targeting students in grades 1‐6 that seeks to reduce childhood risk behaviors, including sexual health behaviors. Professional development on this curriculum is being provided to appropriate grade level teachers.

*Say Yes to Education Buffalo*, a district contracted partner organization supported 28 district community schools and aimed to increase high school and postsecondary completion rates*. Say Yes* school facilitators participated on *School Well‐Being Teams*, received professional development on WSCC implementation, and utilized YRBS data to guide their community school programming. The school facilitators assisted with wellness policy implementation, built relationships with, and linked students to appropriate sexual health service providers via the promotion of the *H.O.P.E. Buffalo Pocket Guide*.

## Health Services and Student Support Services

Nurses provided individualized health education and made special accommodations for students with special needs. To assist ENL students with sexual health education, nurses utilized a translations phone line. School nurses assured that students accessing condoms were taught how to use them correctly and they partnered with the Erie County Department of Health to run sexually transmitted infection testing. Nurses, school counselors, psychologists and social workers received professional development on the utilization of the WSCC model, school‐level and district‐level sexual health YRBS data, comprehensive sexuality education and the *H.O.P.E. Buffalo Resource Pocket Guide*. These student support services personnel worked diligently to provide guidance to students on how to access community sexual health services as well as support for pregnant and parenting teens as needed.

## IMPLICATIONS FOR SCHOOL HEALTH

Implementing a comprehensive wellness policy in a large urban setting with over 6000 employees and 34,000 students is challenging. By having a strong written district wellness policy we increased implementation across the district and its schools.^5^, ^6^ Wellness policies should include strong language to indicate that action is required, including words such as shall, will, must, require, all, comply, and enforce.^6^ Wellness policies should also include wording in the health education domain stressing the importance that sexual health education be offered in a developmentally and intellectually appropriate manner, responsive to students and families with disabilities and designed to improve equity and reduce health disparities. Policies should guide health education to address the social determinants of health and provide for the diversified needs of students as well as varied needs of the community.

Ongoing communication, collaboration, and coordination (3Cs) of the policy development and implementation through school *Well‐Being Teams, District Health Committees*, families, and community partners can be realized with a knowledgeable District Wellness Coordinator. Our experience suggests employing a coordinator that has training in educational leadership, education in public/community/school health, and be employed as a certified district administrator to be most effective. Districts should dedicate fiscal resources to hiring a District Wellness Coordinator and programmatic resources to support wellness policy, WSCC model and WSCC organizational structure implementation. Resources should also be allocated for ongoing professional development in team leadership, sexual health instruction, the condom availability program, and YRBS/SHI administration in each school.

Districts should intentionally plan to develop strong partnerships and relationships across numerous WSCC domains, including parents, students, and community partners, that are fostered through trust and transparency. Trust takes time to develop. We met parents where they were at churches, community centers, at parent‐sponsored meetings, and created nonthreatening spaces across the community to hear parent voices. We partnered with the Community Health Worker Network of Buffalo which offered parent training to be a part of the *School Wellness Collaborative* and *School Whole Child Well‐Being Teams*. Implementation of the health education domain of the WSCC model requires perseverance and the ability to work with and around educational and political structures to alter risky student sexual behaviors. Districts should make concerted efforts to include parents of and students with special education needs as well as ENL families when planning for programs, policies, and practices (3Ps). Consideration should be made to incorporate community health workers into WSCC model implementation.

Districts should provide continuing professional development around sexual health education to other supporting domains of the WSCC model (ie, health services, parent and student engagement, counselor, psychologists and social workers, community partners) when striving to make system changes to sexual health education that are sustainable. Moving forward, districts desiring to improve student sexual health behaviors in a sustainable manner need to provide ongoing age and developmentally appropriate sexual health education via an evidenced‐based curriculum. Ongoing, annual professional development for new as well as veteran health teachers is imperative to sexual health curriculum success. Sexual health education should be taught by certified health education teachers who desire to teach health education and who are trained to teach to state and/or national standards reflective of the community.^16^ We strongly encourage schools to administer the YRBS in their district as data drives policy actions; what gets measured gets done in schools.

This case study illustrated how a strong wellness policy, YRBS and SHI data, and ongoing professional development positively altered sexual health behaviors through the WSCC domain of health education and the supporting role of parent and student engagement, community involvement, health services, student support staff (counselor, psychologists and social workers), and community involvement using the 3Ps and 3Cs. Comprehensive sexual health education empowers and protects all students and fosters healthy development, including ENL students and students with special needs. The WSCC organizational structure (Figure 1) provides numerous channels for communication and coordination of district and community resources so that every student will have the sexual health knowledge, attitudes, skills, and behaviors to be healthy.

### Human Subject Approval Statement

Preparation of this paper did not include performing original research requiring inclusion of human subjects.

### Conflict of Interest

The authors declare no conflict of interest.
